# Effects of Dispositional Resilience and Self-Efficacy on Practice in Advanced Care Planning of Terminally Ill Patients among Taiwanese Nurses: A Study Using Path Modeling

**DOI:** 10.3390/ijerph18031236

**Published:** 2021-01-30

**Authors:** Hsueh-Hsing Pan, Li-Fen Wu, Li-Fang Chang, Yu-Chun Hung, Chin Lin, Ching-Liang Ho

**Affiliations:** 1 School of Nursing, National Defense Medical Center, Taipei City 11490, Taiwan; 2 Department of Nursing, Tri-Service General Hospital, Taipei City 11490, Taiwan; wulifen@mail.ndmctsgh.edu.tw (L.-F.W.); fang_niki@mail.ndmctsgh.edu.tw (L.-F.C.); w51@mail.ndmctsgh.edu.tw (Y.-C.H.); 3 Graduate Institute of Medical Sciences, National Defense Medical Center, Taipei City 11490, Taiwan; 4 Graduate Institute of Life Sciences, National Defense Medical Center, Taipei City 11490, Taiwan; xup6fup@mail.ndmctsgh.edu.tw; 5 Division of Hematology and Oncology, Tri-Service General Hospital, National Defense Medical Center, Taipei City 11490, Taiwan; a2241@mail.ndmctsgh.edu.tw

**Keywords:** advance care planning, knowledge, attitude, practice, path modeling, dispositional resilience, self-efficacy, terminally ill patients

## Abstract

This study aimed to expand on previous research elucidating the effects of dispositional resilience and self-efficacy on practice in advanced care planning (ACP) of terminally ill patients among Taiwanese nurses using path modeling. This cross-sectional study was conducted using cluster sampling. Data were collected using demographics, nurses’ knowledge, attitude, and practice of ACP (KAP-ACP) inventory, Dispositional Resilience Scale, and General Self-Efficacy Scale. A total of 266 nurses from a tertiary medical center in northern Taiwan participated in this study in 2019. The results showed that gender and ward were significant K-ACP predictors among nurses. The ACP knowledge, ward, and experience of caring for terminally ill friends or relatives were significant A-ACP predictors, whereas ACP attitudes, dispositional resilience, self-efficacy, ward, and the frequency of caring for terminally ill patients were the key predictors of P-ACP. The path modeling showed that dispositional resilience; self-efficacy; medical, surgical, hematology and oncology wards; previous experience in caring for terminally ill friends or relatives; participating in the do-not-resuscitate signature; and the frequency of caring for terminally ill patients directly influenced ACP practices. We recommend that nurses enhance their dispositional resilience and self-efficacy, which may encourage them to appreciate the value of ACP practice of terminally ill patients and improve the quality of care.

## 1. Introduction

Due to advancements in medical treatment, people’s life expectancy has been prolonged. However, when facing the terminal stage of a disease in their patients, medical staff may apply active medical treatment due to family pressure, which enables patients to stay alive and prolong the process of dying due to the use of life support medical interventions. In this process, patients often experience a lot of physical and psychological pain [1]. Under the influence of Chinese traditional culture, most Taiwanese do not talk about death, which is considered a taboo. Thus, patients cannot fully express their wishes while still aware and awake. Consequently, their family members often make medical decisions in the terminal stage, rather than the patients themselves, which leads to regrets that patients could not have a good death [1].

Taiwan passed the Patient Right to Autonomy Act by Legislative Yuan on 18 December 2016, which has been implemented since 6 January 2018. The Act aims to ensure patients’ medical autonomy, protect their rights and interests in a good death, and promote a harmonious relationship between doctors and patients. The provisions clearly state that patients have the right to be informed about their condition and the possible effect of medical options and their prognosis, as well as the right to choose and decide on the medical options provided by doctors [2].

Advance care planning (ACP) emphasizes the discussion on the medical treatment or death of terminally ill patients, as well as the needs and expectations of patients pertaining to future medical treatment. Medical staff discuss with patients their medical wishes, treatment goals, and expectations of care places in a structured way to avoid unnecessary medical intervention and hospitalization. In addition, the ACP discussion increases hospice care utilization and promotes emotional expression among family members, which, in turn, improves the quality of end-of-life care for patients [3].

Nurses play an important role in assisting and endorsing decisions about end-of-life concerns for terminally ill patients and their families. In the process of actively promoting ACP in keeping with the government’s recommendation, the knowledge, attitude, and practice of ACP (KAP-ACP) of terminally ill patients by nursing staff are major determinants when engaging in consequential end-of-life discussions with patients to promote ACP success. The better the nurses’ knowledge and attitude of ACP, the better they can introduce ACP to patients at an appropriate time and help them to formulate the ACP content as soon as possible [4]. Studies have shown that early ACP discussions with patients can help patients to get the medical care they want, reduce invalid medical treatment, and improve the satisfaction with hospice care service and medical care of patients and their families. It can also reduce the pressure and anxiety of family members facing the medical treatment and decision-making process of terminally ill patients, and improve the quality of life [5,6,7]. Prior studies have investigated KAP-ACP behaviors of nurses [1,8,9,10]. However, some of these studies focused on the population of oncology or emergency nurses, and most of them explored the related factors of KAP-ACP. Moreover, multiple regression analysis plays an important role in providing evidence about factors that predict nurses’ KAP-ACP. Path modeling is advantageous in that it analyzes direct and indirect properties simultaneously, with numerous independent and dependent variables. In addition, it enables the exploration of causal relationships among a set of variables [11]. Thus, the present study aimed to investigate the causal relationship between KAP-ACP of terminally ill patients using path modeling among Taiwanese nurses.

## 2. Methods

### 2.1. Study Design and Population

This study was conducted at a tertiary medical center in northern Taiwan between June and August 2020, using cross-sectional design. Regarding the recruitment of nurses, cluster sampling was performed by wards and intensive care units (ICUs). A sample size of 240 was expected using a G* power of 3.1. A two-tailed *p*-value was set at 0.05, whereas power was set at 0.95, the effect size at 0.15, and predictor at 20. Additionally, considering a 20% attrition rate, 288 participants were recruited in total. Finally, 22 participants discontinued participation, and 266 participants completed the study. The effective sample to this questionnaire were the following criteria: being a registered nurse, the ability to speak Mandarin, and consent to join this study.

### 2.2. Instruments

#### 2.2.1. Demographics and Work-Related Characteristics

Demographic characteristics such as age, gender, education level (Junior college, Bachelor’s degree, or Master’s degree), practicing religion (no or yes), and marital status (single or married) were included. Work-related characteristics were listed as (1) nurse’s service ward [hematology and oncology (HemaOnco), medical, surgical, or ICU], (2) service duration in nursing, (3) experience with caring for terminally ill friends or relatives (yes or no), (4) frequency of caring for terminally ill patients (rated on a 1–5 scale, with 1 indicating “never” and 5 indicating “always”), (5) participation in do-not-resuscitate (DNR) signature (yes or no), and (6) receiving ACP-related education (yes or no).

#### 2.2.2. Nurses’ Knowledge, Attitude, and Practice of ACP Inventory

The nurses’ KAP-ACP inventory was developed based on literature review and the Patient Right to Autonomy Act (Laws and Regulations Database of The Republic of China, 2020). It includes three scales measuring the knowledge (K-ACP), attitude (A-ACP), and practice (P-ACP) of ACP of terminally ill patients among nurses. The K-ACP comprises 10 items rated as “true,” “false,” or “I don’t understand.” It assesses the level of understanding of the concept, purpose, and significance of ACP of terminally ill patients among nurses. The proportion of correct answers is calculated by dividing the number of correct responses by the total number of questions. The A-ACP comprises 15 items assessing nurses’ attitudes regarding ACP of terminally ill patients. All items are ranked on a 5-point Likert -scale ranging from 1 to 5. A score of 1 indicates “strongly disagree” and 5 indicates “strongly agree.” Items 8, 9, 10, 11, 13, 14, and 15 are negatively worded, and are therefore reverse scored. The total score ranges from 15 to 75, with a higher score indicating a better attitude toward ACP of terminally ill patients. The P-ACP comprises 10 items that assess the nurse’s ability to talk proactively with a patient or family members regarding ACP of terminally ill patients. Item 9 is negatively worded, and is therefore reverse scored. Items are rated on a 1–5 scale, with 1 representing “never” and 5 representing “always”. The total score ranges from 10 to 50, with a higher score indicating better practice of ACP of terminally ill patients among nurses.

The content validity index (CVI) for this tool was established by a panel of five experts in the field of end-of-life care, which included two professors, two physicians, and a hospice combined care nurse. These experts evaluated the CVI of the KAP-ACP inventory based on relevance, accuracy, and applicability, each rated on a 1–5 scale. The CVI was computed as number of experts who graded an item as 4 or 5, divided by the total number of experts. The CVI in this study was 0.90. A pilot study recruited 15 nurses, and the Cronbach’s α reliability of the K-ACP, A-ACP, and P-ACP of terminally ill patients among nurses was 0.502, 0.908, and 0.901, respectively. For the present sample of 266 nurses, the Cronbach’s α reliability of the K-ACP, A-ACP, and P-ACP was 0.558, 0.832, and 0.882, respectively.

#### 2.2.3. Dispositional Resilience Scale

The Dispositional Resilience Scale (DRS) was validated using the Chinese version by Wong, et al. [12]. It assesses psychological hardiness, which is the unique style that distinguishes individuals under stress. It is assessed with reference to commitment toward life, control of life, and readiness to beat challenges. This tool comprises 15 items across three subscales, including commitment, control, and challenge. Each subscale contains 5 items that are ranked on a 0–3 scale, with 0 representing “disagree” and 3 representing “totally agree.” The total score ranges from 0 to 45, with a higher score showing greater hardiness. This scale exhibited satisfactory reliability and validity in a previous study [12]. With reference to internal consistency, the Cronbach’s *α* of the overall DRS in this study was 0.865, and that for the subscales of commitment, control, and challenge was 0.808, 0.817, and 0.502, respectively, for a sample of 266 nurses.

#### 2.2.4. General Self-Efficacy Scale

The General Self-Efficacy Scale (GSES) is a 20-item tool developed in 1981, which was later modified into a 10-item shortened version [13]. This scale assesses a wide and constant sense of personal ability to manage a variety of stressful conditions efficiently. The Chinese version was unidimensional, with an internal reliability (Cronbach’s α) of 0.92. It contains 10 items ranked on a 1–4 scale, with 1 being “completely incorrect” and 4 being “completely correct.” The total score ranges from 10 to 40, with higher scores indicating greater self-efficacy to deal with stressful conditions. With reference to internal consistency, the Cronbach’s *α* of the GSES was 0.936 for the 266 nurses in the present study.

### 2.3. Study Process

Before this study implementation, a pilot study recruited 15 nurses to examine the feasibility of this study using convenience sampling. Nurses were recruited using cluster sampling by wards and ICUs. The researcher described the aims, methods, and process of this study at ward meetings. Questionnaires were anonymous, and the information was kept confidential. It took 15–20 min for participants to complete the questionnaires. Furthermore, they were allowed to discontinue participation any time during the study, at their discretion. A total of 266 participants completed the questionnaires. All study processes were approved by the Institutional Review Board (IRB 1-108-05-151).

### 2.4. Statistical Analysis

**Figure 1:**
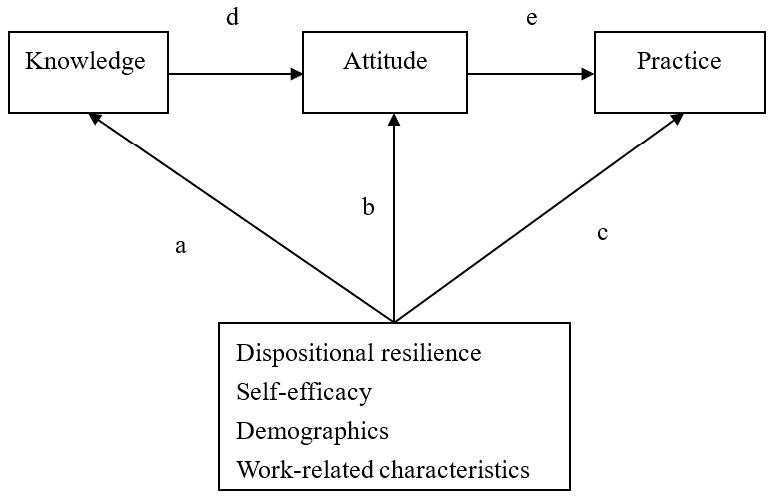
Path modeling. (**a**. the standard coefficients of covariates to knowledge; **b**. the standard coefficients of covariates to attitude; **c**. the standard coefficients of covariates to practice; **d**. the standard coefficient of knowledge to attitude; **e**. the standard coefficient of attitude to practice).

Data were entered into Microsoft Excel and were then analyzed using IBM SPSS Statistics for Windows, version 22.0 (IBM Corp., Armonk, NY, USA). Frequency and percentage were used to describe categorical variables, and mean and standardized deviation (SD) were used to describe continuous variables. Multiple linear regression was performed to explore the predictors of nurses’ KAP-ACP scores. Path modeling was utilized to assess the direct or indirect relationships among a set of variables, including dispositional resilience, self-efficacy, demographics, and work-related characteristics. Figure 1 presents the path model tested in this study. A *p*-value < 0.05 was regarded as statistically significant.

## 3. Results

### 3.1. Demographics and Work-Related Characteristics

Table 1 shows the demographics and work-related characteristics of the study participants. A total of 266 participants completed the study, the mean age was 30.1 ± 7.7 years and up to 91% were women. Most participants had a bachelor’s degree (79.3%) and were single (82.7%), and almost equal numbers reported having a religious belief (53.0%) and not having a religious belief (47.0%). Almost half (45.5%) of the participants worked at the ICU, 64.7% of the participants had no experience in caring for terminally ill friends or relatives, 82.3% had participated in DNR signature, and 64.3% had received education related to ACP. The participants’ mean service duration in nursing was 7.2 ± 6.9 years, and the mean frequency of caring for terminally ill patients was 2.8 ± 1.1 times.

### 3.2. Knowledge, Attitude, and Practice toward ACP of Terminally Ill Patients, Dispositional Resilience, and Self-Efficacy among Nurses

Nurses’ knowledge, attitude, and practice toward ACP of terminally ill patients, dispositional resilience, and self-efficacy have been presented in Table 2. The mean proportion of correct answers on ACP knowledge was 91.5%, while the mean scores on ACP attitude and practice were 60.3 ± 7.7 and 34.9 ± 8.2, respectively. The mean scores on dispositional resilience and self-efficacy were 27.6 ± 4.3 and 26.3 ± 5.4, respectively. Among the subscales of dispositional resilience, nurses scored the highest on the control subscale and lowest on the challenge subscale.

### 3.3. Predictors of KAP-ACP of Terminally Ill Patients among Nurses

Table 3 presents the predictors of nurses’ ACP knowledge, attitude, and practice of terminally ill patients. After adjusting for potential confounders, female nurses had a higher mean score on the K-ACP of terminally ill patients than did male nurses (β = 0.49, 95% confidence interval [CI] = 0.14–0.83, *p* < 0.01), and those who worked in the medical ward had a higher mean score on the K-ACP of terminally ill patients than did those who worked in the ICU (β = 0.46, 95% CI = 0.18–0.73, *p* < 0.001).

Further, nurses with a higher K-ACP of terminally ill patients score had a higher A-ACP of terminally ill patients score (β = 2.32, 95% CI = 1.25–3.38, *p* < 0.001). Nurses who worked at the hematology and oncology wards (β = 3.61, 95% CI = 0.37–6.86, *p* < 0.05), and the medical ward (β = 2.86, 95% CI = 0.28–5.49, *p* < 0.05) had a higher mean score on the A-ACP of terminally ill patients than did those who worked in the ICU. Nurses with experience in caring for terminally ill friends or relatives had a higher mean score on the A-ACP of terminally ill patients than did those without such experience (β = 2.20, 95% CI = 0.11–−4.29, *p* < 0.05).

Finally, nurses with a higher score on the A-ACP of terminally ill patients (β = 0.21, 95% CI = 0.10–0.32, *p* < 0.001), dispositional resilience (β = 0.36, 95% CI = 0.14–0.58, *p* < 0.01), and self-efficacy (β = 0.28, 95% CI = 0.10–0.47, *p* < 0.01), and those with a higher frequency of caring for terminal patients (β = 1.91, 95% CI = 1.16–2.67, *p* < 0.001) had a higher score on the *p*-ACP of terminally ill patients. Additionally, those who worked at the hematology and oncology ward had a higher mean score on the *p*-ACP of terminally ill patients (β = 3.98, 95% CI = 1.21–6.74, *p* < 0.01), but those who worked at the surgical ward had a lower mean score on the A-ACP of terminally ill patients (β = −2.85, 95% CI = −4.92–−0.78, *p* < 0.01) as compared to those who worked at the ICU.

### 3.4. Path Modeling of KAP-ACP among Nurses

We used path modeling to examine the causal relationships between KAP-ACP of terminally ill patients and dispositional resilience, self-efficacy, demographics, and work-related characteristics. These findings have been presented in Table 4. Dispositional resilience (Coefficient = 0.354, *p* < 0.001), self-efficacy (Coefficient = 0.337, *p* < 0.001), and frequency of caring for terminally ill patients (Coefficient = 0.328, *p* < 0.001) had a direct positive relationship with the P-ACP of terminally ill patients score based on the standardized coefficient estimates for the paths. Additionally, working in the medical (Coefficient = 0.187, *p* = 0.003), surgical (Coefficient = 0.165, *p* = 0.008), or hematology or oncology ward (Coefficient = 0.229, *p* < 0.001) had a direct positive relationship with the P-ACP of terminally ill patients score as compared to working in the ICU. Further, as compared to never having cared for terminally ill friends or relatives and not having participated in DNR signature, past experience of caring for terminally ill friends or relatives (Coefficient = 0.170, *p* = 0.006) and participating in DNR signature (Coefficient = 0.139, *p* = 0.025) had a direct positive correlation with the P-ACP of terminally ill patients score.

## 4. Discussion

### 4.1. Factors Affecting the KAP-ACP of Terminally Ill Patients among Nurses

Although the number of female nurses in the study was far more than male nurses, the results of this study showed that being a female nurse and working at a medical ward were positive predictors of ACP knowledge of terminally ill patients among nurses. A study in Saudi Arabia showed that women had better knowledge on advanced directives for patients with cancer than their male counterparts [14]. In the previous study considering learning motivation, women had a higher level of autonomy in learning motivation than men, that is, women had better motivation to learn independently, and thus, they could take the initiative to learn to maintain their professional knowledge [15,16]. Nurses working at a medical ward had better knowledge regarding the ACP of terminally ill patients. This finding was similar with that of prior studies [9,17,18,19]. Nurses working in different wards still need basic skill in caring for patients. Nurses working in ICUs dominated critical care for patients with life-threatening conditions. They also gave patients the most effective treatment, avoided deterioration of the condition, and reduced the number of deaths [20,21]. However, medical nurses emphasized delivering holistic care comprising physical, psychological, social, and spiritual care. They had higher levels of ACP awareness because they regularly received ACP-related training [10,19].

This study found that nurses with ACP knowledge of terminally ill patients had better attitudes toward the ACP of terminally ill patients. A prior study conducted in Taiwan showed that the better the knowledge of ACP among nurses, the more positive attitude toward ACP [8]. Nurses with ACP knowledge would have greater confidence to enter an ACP discussion with a positive attitude. This would also enable them to talk keenly with patients and their families, to support them to make appropriate decisions, and to propose appropriate end-of-life care [22]. Nurses who worked at hematology, oncology, and medical wards had better attitudes toward ACP than those who worked at the ICU. Nurses who worked at hematology and oncology had more opportunities to receive ACP-related education or training courses than those who worked at other wards or ICUs [18]. Therefore, those nurses have better knowledge and attitudes toward ACP of terminally ill patients. Nurses who had experience on caring for terminally ill friends or relatives had better attitudes toward the ACP of terminally ill patients. This finding is similar to that of prior studies [18,23]. Nurses who have experienced the deaths of relatives were more willing to discuss ACP with a positive attitude when caring for terminally ill patients. They could understand the family members and were able to better appreciate the patient’s wishes, values, and end-of-life care decisions [24].

Our results showed that nurses with better ACP attitudes also had better ACP practice with terminally ill patients. Many previous studies have shown similar results [8,10,25]. Attitudes refer to the participant’s feelings about the problem, while practice is the way of expressing an attitude. Nurses with positive attitudes can confront professional challenges and enhance professional understanding, competence, and satisfaction [26]. Our results showed that self-efficacy might affect practice toward the ACP of terminally ill patients among nurses. This finding is consistent with those reported by prior studies [27,28,29]. Self-efficacy helps nurses feel more confident about their ability to provide appropriate, timely, and tender care. Nurses with higher self-efficacy can promote ACP discussions with patients [30,31]. In addition, nurses with better dispositional resilience tend to show better ACP practice among nurses. Various studies have shown that dispositional resilience was correlated with practice [25,32,33,34]. Dispositional resilience is a kind of hardiness, a personality trait regarding how people cope with stressful events [12]. It can help nurses confront stress and overcome challenges [32,33,35]. Nurses working at hematology and oncology wards showed a better ACP practice of terminally ill patients. As we know, nurses working at hematology and oncology wards have more opportunities to receive ACP-related education or training courses [18]. They are also encouraged to explain the patients’ prognoses [19]. Therefore, nurses working at hematology and oncology wards have better ACP knowledge of terminally ill patients and confidence to discuss ACP with patients in various types of end-of-life care. Nurses who care more frequently for terminally ill patients had better ACP practice. ACP involves healthcare professionals and patients, and it aims to enable patients to express their wishes as their disease progresses. Nurses who often care for dying patients tended to have an increased ACP knowledge [36]. In addition, they may be more aware of the importance of ACP discussions because they increasingly face the dying process and a shortened lifespan [37]. Therefore, these nurses have more opportunities to discuss ACP with the families of terminally ill patients.

### 4.2. Path Modeling of KAP-ACP of Terminally Ill Patients among Nurses

Path modeling is a method to determine causal relationships among a set of variables [11]. The results of path modeling revealed that dispositional resilience, self-efficacy; medical, surgical, and hematology and oncology wards; caring for terminally ill friends or relatives; participation in the DNR signature; and the frequency of caring for terminally ill patients had direct positive effects on the ACP liaison.

Dispositional resilience refers to the individual’s internal ability to actively overcome difficulties in the clinical environment and cope with the stress environment, which can help nurses cope with the situation and reduce psychological distress. It is also a required ability of nurses to show professionalism when helping patients. Dispositional resilience is not only a happy indicator, but also a process of keeping individuals healthy or helping them to recover quickly after adversity [33,38]. The association between self-efficacy and practice is an important finding. Self-efficacy is characterized as a personal belief in one’s ability to execute specific skills. It can help nurses enhance their belief that they can perform a task or take action. Self-efficacy is helpful to practice and can improve the ACP implementation [28,29,30].

Nurses who worked in medical, surgical, hematology, and oncology wards were influenced more by ACP in their practice than those who worked in the ICU. This finding is related to that of prior studies [10,22,39]. It is likely that nurses working in the ward had more opportunities to talk about ACP with patients and their families in their daily practice than those working in the ICU. Caring for terminally ill friends or relatives and higher frequency of caring for terminally ill patients also directly affected the ACP practice. These results are comparable to those of previous studies [37,40]. Nurses have experienced life-threatening situations for patients, friends, or relatives, so they did not choose active or life-sustaining treatments when the patients were supposed to die within a few weeks. Therefore, nurses who had more experience with or who had more frequently cared for terminally ill patients, friends, or relatives also had a better understanding of patients’ illness and the importance of discussing ACP [40].

Participation in the DNR signature directly affected nurses’ ACP practice of terminally ill patients. A DNR is authorized to avoid patients from being subject to invalid treatment procedures, indicating that patients only prefer to receive comfort care at the terminal stage or near death [41]. Nurses play a dominant role in the medical decision-making process, and the DNR discussion offers recommendations for terminally ill patients and their family members, which enables them to make medical decisions that facilitate the provision of better care. Therefore, nurses who participate in the DNR signature are aware of the ACP conversation with patients and their families to have comfort care for patients in the terminal stage.

### 4.3. Limitations and Strengths

This study considered several demographics, work-related characteristics, dispositional resilience, and self-efficacy in the multiple linear regression model to minimize the potential confounding. In addition, path modeling was utilized to assess the direct or indirect relationships among a set of variables of KAP-ACP of terminally ill patients among nurses. Despite the above strengths, there are some limitations to this study. First, only the nurses from one medical center participated in this study, limiting the generalizability of our findings. Future studies can recruit samples in different centers to confirm our findings. Second, this study used a cross-sectional design, and the trend of KAP toward ACP over time in nurses was not explored. Future studies can use a follow-up design to discover the change in the KAP-ACP among nurses. Third, we used a self-reporting questionnaire to examine KAP-ACP. Future studies are required to use more objective measurement tools and calculate the frequency of the ACP discussion to determine the ACP practice level among nurses.

## 5. Conclusions

This study suggests that female nurses and nurses who work at hematologic, oncology, and medical wards had better ACP knowledge of terminally ill patients. Nurses who had better ACP knowledge of terminally ill patients; worked at hematologic, oncology, and medical wards; and experienced caring for terminally ill friends or relatives had a better attitude toward ACP of terminally ill patients. Nurses who had better ACP attitudes of terminally ill patients; showed dispositional resilience and self-efficacy; worked at hematologic and oncology wards; and cared more frequently for terminally ill patients also had better ACP practices. Additionally, dispositional resilience; self-efficacy; medical, surgical, hematologic, and oncology wards; experience in caring for terminally ill friends or relatives, participation in the DNR signature, and the frequency of caring for terminally ill patients directly influenced the ACP practice of terminally ill patients. Based our findings, we recommend that nurses enhance their self-efficacy and dispositional resilience by some training programs, such as ACP education or mindfulness-based education, which may enable them to understand the value of ACP practice and promote the quality of patient care.

## Figures and Tables

**Table 1:** Demographics and work-related characteristics (N = 266).

Variable	N (%) /Mean ± SD
**Demographics**	
Age(year)	30.1 ± 7.7
Gender	
Female	242 (91.0%)
Male	24 (9.0%)
Education level	
Junior college	34 (12.8%)
Bachelor	211 (79.3%)
Master	21 (7.9%)
Religious Belief	
No	125 (47.0%)
Yes	141 (53.0%)
Marital Status	
Single	220 (82.7%)
Married	46 (17.3%)
**Work-related characteristics**	
Ward	
HemaOnco	31 (11.7%)
Medical	53 (19.9%)
Surgical	61 (22.9%)
ICU	121 (45.5%)
Service duration in nursing (year)	7.2 ± 6.9
Caring for terminally ill friends or relatives	
No	172 (64.7%)
Yes	94 (35.3%)
Frequency of caring for terminally ill patients	2.8 ± 1.1
Participation in DNR signature	
No	219 (82.3%)
Yes	47 (17.7%)
Receiving education related to ACP	
No	95 (35.7%)
Yes	171 (64.3%)

SD standard deviation; HemaOnco Hematologic and oncology; ICU Intensive care unit; DNR do not resuscitate; ACP Advance care planning.

**Table 2:** Knowledge, attitude, and practice toward ACP of terminally ill patients, dispositional resilience, and self-efficacy among nurses (N = 266).

Variable	Mean ± SD
**Knowledge**	9.2 ± 0.8
Proportion of correct answer (%)	91.5 ± 8.4
**Attitude**	60.3 ± 7.7
**Practice**	34.9 ± 8.2
**Dispositional resilience**	27.6 ± 4.3
Commitment	8.3 ± 1.7
Control	10.0 ± 1.8
Challenge	9.3 ± 1.7
**Self-efficacy**	26.3 ± 5.4

ACP Advance Care Planning; SD standard deviation.

**Table 3:** Predictors of knowledge, attitude, and practice of ACP of terminally ill patients among nurses (N = 266).

	Knowledge	Attitude	Practice
Variables	β (95%CI)	β (95%CI)	β (95%CI)
**Knowledge**	-	2.32 *** (1.25~3.38)	0.69 (−0.31~1.68)
**Attitude**	-	-	0.21 *** (0.10~0.32)
**Practice**	-	-	-
**Dispositional resilience**	0.01 (−0.02~0.04)	0.11 (−0.14~0.35)	0.36 ** (0.14~0.58)
**Self-efficacy**	−0.01 (−0.03~0.02)	−0.03 (−0.23~0.18)	0.28 ** (0.10~0.47)
**Demographics**			
Age (years)	−0.002 (−0.02~0.01)	0.04 (−0.10~0.18)	−0.10 (−0.23~0.02)
Gender			
Female	0.49 ** (0.14~0.83)	−2.69 (−5.81~0.43)	1.64 (−1.19~4.47)
Male	Reference	Reference	Reference
**Education level**			
Junior college	−0.12 (−0.63~0.39)	−0.81 (−5.30~3.68)	−1.62 (−5.67~2.43)
Bachelor	−0.25 (−0.68~0.19)	−0.55 (−4.40~3.29)	−2.50 (−5.97~0.97)
Master	Reference	Reference	Reference
**Religious belief**			
No	Reference	Reference	Reference
Yes	0.02 (−0.19~0.23)	1.50 (−0.34~3.34)	0.40 (−1.28~2.07)
**Marital status**			
Single	Reference	Reference	Reference
Married	−0.09 (−0.39~0.21)	−1.38 (−4.03~1.27)	−0.20 (−2.60~2.20)
**Work-related characteristics**			
**Ward**			
HemaOnco	0.27 (−0.05~0.58)	3.61 * (0.37~6.86)	3.98 ** (1.21~6.74)
Medical	0.46 *** (0.18~0.73)	2.86 * (0.28~5.49)	0.11 (−2.15~2.37)
Surgical	−0.06 (−0.32~0.20)	−0.10 (−2.50~2.29)	−2.85 ** (−4.92~−0.78)
ICU	Reference	Reference	Reference
**Service duration in nursing**	0.001 (−0.01~0.02)	0.02 (−0.11~0.16)	0.02 (−0.11~0.16)
**Caring for terminally ill friends or relatives**			
No	Reference	Reference	Reference
Yes	0.01 (−0.23~0.25)	2.20 * (0.11~4.29)	0.48 (−1.43~2.39)
**Frequency of caring for terminally ill patients**	−0.01 (−0.10~0.09)	0.26 (−0.58~1.10)	1.91 *** (1.16~2.67)
**Participation in DNR signature**			
No	Reference	Reference	Reference
Yes	−0.03 (−0.32~0.26)	−0.45 (−3.00~2.11)	0.32 (−1.98~2.63)
**Receiving education related to ACP**			
No	Reference	Reference	Reference
Yes	0.01 (−0.29~0.30)	−0.38 (−2.98~2.22)	−1.14 (−3.50~1.21)

HemaOnco hematologic and oncology; ICU intensive care unit; DNR do-not-resuscitate; ACP Advance care planning; CI confidence interval; * *p* < 0.05; ** *p* < 0.01; *** *p* < 0.001.

**Table 4:** Path modeling for knowledge, attitude, and practice of ACP of terminally ill patients among nurses (N = 266).

	Knowledge→Attitude→Practice		Attitude→Practice		Practice	
Variables	Coefficients	*p* Value	Coefficients	*p* Value	Coefficients	*p* Value
**Dispositional Resilience**	0.008	0.902	0.087	0.159	0.354	<0.001
**Self-efficacy**	0.008	0.895	0.091	0.139	0.337	<0.001
**Demographics**						
Age	−0.002	0.979	−0.017	0.787	−0.049	0.427
Gender						
Male/Female	−0.001	0.993	−0.011	0.853	−0.017	0.783
Education level						
Junior college/Master	0.002	0.968	0.028	0.649	0.098	0.113
Bachelor/Master	−0.002	0.975	−0.024	0.693	−0.092	0.136
Religious Belief						
Yes/No	0.000	0.999	0.000	0.996	0.027	0.661
Marital Status						
Married/Single	−0.002	0.976	−0.020	0.750	−0.086	0.163
**Work-related characteristics**						
Ward						
HemaOnco/ICU	−0.004	0.944	−0.059	0.339	0.229	<0.001
Medical/ICU	0.003	0.958	0.043	0.488	0.187	0.003
Surgical/ICU	0.003	0.966	0.037	0.553	0.165	0.008
Service duration in nursing	−0.003	0.960	−0.033	0.589	−0.102	0.099
Caring for terminally ill friends or relatives						
Yes/No	0.004	0.953	0.038	0.536	0.170	0.006
Participation in DNR signature						
Yes/No	0.004	0.954	0.038	0.534	0.139	0.025
Frequency of caring for terminally ill patients	0.008	0.891	0.089	0.151	0.328	<0.001
Receiving education related to ACP						
Yes/No	0.002	0.969	0.028	0.651	0.094	0.128

HemaOnco hematologic and oncology; ICU intensive care unit; DNR Do Not Resuscitate; ACP Advance care planning.

## Data Availability

The data presented in this study are available on request from the corresponding author. The data are not publicly available due to privacy.
